# Toll-Like Receptors in Hepatic Ischemia/Reperfusion and Transplantation

**DOI:** 10.1155/2010/537263

**Published:** 2010-08-05

**Authors:** John Evankovich, Timothy Billiar, Allan Tsung

**Affiliations:** Department of Surgery, University of Pittsburgh School of Medicine, 200 Lothrop St., Pittsburgh, PA 15213, USA

## Abstract

The family of Toll-like receptors (TLRs) function as pattern-recognition receptors (PRRs) that respond to a myriad of highly conserved ligands. These substrates include pathogen-associated molecular patterns (PAMPs) for the recognition of invading pathogens, as well as damage-associated molecular patterns (DAMPs) for the recognition of endogenous tissue injury. While the functions of TLRs are diverse, they have received much attention for their roles in ischemia/reperfusion (I/R) injury of the liver and other organs. The TLRs play central roles in sensing tissue damage and activating the innate immune system following I/R. Engagement of TLRs by endogenous DAMPs activates proinflammatory signaling pathways leading to the production of cytokines, chemokines and further release of endogenous danger signals. This paper focuses on the most recent findings regarding TLR family members in hepatic I/R injury and transplantation.

## 1. Introduction

The liver is a central integrator of the systemic immune response following acute traumatic or surgical insults. It is subject to injury and dysfunction following local insults such as ischemia-reperfusion (I/R), as well as systemic insults including hemorrhagic shock. Liver I/R is a pathophysiologic process whereby hypoxic organ damage is accentuated following return of blood flow and oxygen delivery. This process involves activation of the innate immune system, causing a proinflammatory response at the site of injury. Although the distal cascade of inflammatory responses resulting in organ damage after I/R injury has been studied extensively, the process by which initial cellular injury after an ischemic insult contributes to activation of the inflammatory response is poorly understood. Recently, Toll-like receptors (TLRs) have been shown to be critical for the full induction of the inflammatory response observed in experimental ischemia and reperfusion. The TLR receptors involved in alerting the innate immune system appear to be activated by damage-associated molecular pattern molecules (DAMPs) that are released during ischemic stress. In this paper, we will summarize the most recent findings regarding the role of TLRs in liver I/R.

## 2. Toll-Like Receptors

The family of Toll-like receptors are important components of the innate immune system responsible for recognizing a variety of exogenous and endogenous molecules [1]. In 1996 it was demonstrated that the *Drosophila* Toll protein is an essential part of the immune response to fungal infection in adult flies in addition to its established role in development [2]. The identification and characterization of the human Toll homologues soon followed [3]. A total of 13 TLRs have been identified in mammals: humans have 10 and mice 12 [4]. While all TLRs are transmembrane proteins, some reside at the cell surface, and some reside intracellularly. TLR1, TLR2, TLR4, and TLR6 are found at the cell surface, and all have an extracellular component comprised of luecine-rich repeat (LRR) domains. TLR3, TLR7, TLR8, and TLR9 are intracellular, primarily located in the endoplasmic reticulum. All TLRs contain a conserved cytoplasmic Toll/IL-1 Receptor (TIR) domain that is shared by the receptors of the IL-1 and IL-18 families [5]. These features allow TLRs to signal through a group of adaptor molecules which also contain TIR domains. TLRs form heterodimers (TLR1 with TLR2 and TLR2 with TLR6, e.g.), or homodimerize (TLR4 and TLR9), and undergo conformational changes after ligand engagement which leads to association of individual TIR domains. Adaptor molecules are then recruited; these include MyD88, MyD88-adaptor-like (MAL, also referred to as TIR domain-containing adaptor protein (TIRAP)), TIR domain-containing adaptor-inducing IFN-*β* (TRIF), TRIF-related adaptor molecule (TRAM), and sterile *α*- and armadillo motif-containing protein (SARM) [6]. Most TLRs utilize MyD88 to initiate intracellular signaling. The exceptions are TLR3, which interacts with TRIF exclusively, and TLR4 which is capable of utilizing both MyD88 and TRIF. MyD88 recruitment initiates activation of additional intermediate signaling molecules; these proteins include IL-1R-associated kinase 4 (IRAK4), which phosphorylates IRAK1, leading to the recruitment of TNFR-associated factor 6 (TRAF6) and TGF-*β*-activated kinase 1 (TAK1). These events culminate in activation of mitogen-activated protein kinases p38, c-Jun N-terminal kinase (JNK), and NF-*κ*B through phosphorylation of I-*κ*K. In contrast to MyD88-dependent signaling, the TRIF-dependent pathway recruits TRAF3, ultimately resulting in transcription of interferon-regulator 3 (IRF3) and production of IFN-*β*. While the MyD88 and TRIF-dependent signaling pathways are distinct, significant overlaps exist. For example, TLR4 signaling through TRIF can result in NF-*κ*B activation, and signaling through MyD88 can induce activation of IRFs, particularly IRF1. 

TLRs are expressed on several different cell types in the liver, including both parenchymal cells and nonparenchymal cells. Hepatocytes express low levels of TLR2, TLR3, TLR4, and TLR5 and are capable of responding to TLR2 and TLR4 ligands [7]. Similarly, biliary epithelial cells express TLR2, TLR3, TLR4, and TLR5. Kupffer cells, the liver's resident macrophages, are critical in the pathogenesis of I/R and express a number of different TLRs. Studies suggest that TLR4 signaling is critical in Kupffer cells because they are the first to be exposed to gut-derived endotoxin. When exposed to physiological levels of LPS, Kupffer cells secrete anti-inflammatory IL-10, suppressing activation of surrounding immune cells [8–10]. In addition to TLR4, Kupffer cells also express TLR2, TLR3, and TLR9. Most other hepatic nonparenchymal cells express various TLRs. Hepatic stellate cells express TLR4 and TLR9, sinusoidal epithelial cells express TLR4, and subsets of hepatic dendritic cells express TLR2, TLR3, TLR4, TLR7, and TLR9. The liver contains a high concentration of natural killer (NK) cells, and they express TLR1, TLR2, TLR4, TLR7, and TLR9 [11, 12]. It is worth noting that an exhaustive analysis of the functional expression of pattern recognition receptors on liver cell types has not been reported. However, we can conclude that activation of TLRs during I/R creates a diverse response in different cell types that respond to different TLR ligands.

## 3. Liver I/R

Ischemia/reperfusion injury is a phenomenon whereby tissues experience damage as a result of temporarily interrupted blood flow (ischemia) followed by its restoration (reperfusion). Clinically, liver I/R occurs in settings of elective liver surgery, trauma, shock, and transplantation. Two categories of hepatic I/R—warm and cold—are similar yet distinct processes that share a number of characteristics, both of which ultimately result in end-organ damage. Warm I/R commonly occurs during surgery, trauma, and low-flow states while cold I/R is experienced during organ preservation prior to transplantation. The pathophysiology of liver I/R injury includes both direct cellular damage as a result of the ischemic insult as well as delayed dysfunction following reperfusion resulting from activation of the immune system. The distal interacting elements in the cascade of inflammatory responses resulting in organ damage following hepatic I/R injury have been extensively studied. However, proximal events that initiate damage during I/R are less well characterized. The most recent work in this field points to a critical role for activation of TLRs after I/R as initiating events in the pathogenesis of I/R injury. 

### 3.1. Warm Ischemia Reperfusion Injury

The process of warm I/R injury involves activation of immune pathways and is dominated by hepatocellular injury. While all cells types in the liver are involved in the process, Kupffer cells, the resident macrophages of the liver, are a key cell type involved in the earliest stages of I/R injury. Amongst their many functions, one of the most important is the production of reactive oxygen species (ROS). They were discovered to be an important source of ROS during I/R in the 1980s [13–15]. Normally, ROS production is useful in eliminating circulating pathogens and is the mechanism responsible for the “respiratory burst” observed when these cells are activated. However, excessive ROS after an ischemic insult is detrimental. While damage occurs directly from ischemia-induced oxidant stress on many cell types during the early phase of injury, ROS from Kupffer cells also contributes to the activation of inflammatory pathways that lead to neutrophil accumulation in the liver, resulting in additional, prolonged injury. Thus, Kupffer cell-derived ROS are involved in the pathogenesis of I/R injury through direct oxidant-mediated damage and by augmenting the local activation of proinflammatory pathways. 

Another hallmark of I/R injury is the release of cytokines and chemokines from cells at the site of injury. Kupffer cells, in addition to releasing ROS, are also principally responsible for the release of cytokines during I/R. Both Tumor necrosis factor-alpha (TNF*α*) and Interluekin-1 (IL-1) are released from Kupffer cells within minutes following reperfusion [16, 17] and promote damage through a number of mechanisms. Briefly, these cytokines recruit neutrophils by promoting upregulation of neutrophil integrins and also activate and recruit CD4+ T cells to the site of injury [18–20]. Additionally, they promote the local production of key chemokine molecules which both attract and activate neutrophils [21–23]. Together, these events culminate in hepatocellular damage and death, resulting in elevated serum transaminase levels and organ damage. While the extent of damage is dependent on a number of factors, including severity of the ischemic insult, all of the pathological factors contributing to I/R injury also share the commonality that they are, in part, dependent on TLR signaling. Thus, while multiple signaling networks are responsible for coordinating the inflammatory response during I/R injury, TLRs are critical in initiating and mediating these effects.

### 3.2. Cold Ischemia Reperfusion Injury

Cold I/R occurs in the setting of solid organ transplantation after a donor graft is harvested. Before cold storage, the organ is perfused with a preservation solution and remains ischemic until it is transplanted into the recipient. When the graft is reperfused cold I/R occurs. In contrast to warm I/R, cold I/R is dominated by damage to the sinusoidal endothelial cells and disruption of the microcirculation rather than damage to the hepatocytes. While cold storage times that occur in human transplantation vary greatly, animal studies focus on storage times of up to 18 hours, though some extend cold storage to 24 hours or more. In addition to cold I/R injury, liver transplantation involves additional factors such as immunologic tolerance and rejection [24, 25].

## 4. Role of Endogenous DAMPs in I/R

Acute, ischemic, and sterile tissue injury activates the innate immune system in a way similar to pathogenic infections from microbes, viruses, and fungi. This phenomenon occurs because pattern recognition receptors, including the TLRs, are capable of recognizing both PAMPs and DAMPs, leading to activation of similar downstream signaling cascades. Since the first report that an endogenous molecule, Heat Shock protein 60, could activate TLR4 signaling [26], a number of additional TLR-activating DAMPs have been discovered including hyaluronan, fibrinogen, heparin sulfate, High Mobility Group Box Protein 1 (HMGB1), and DNA [1]. During I/R, DAMPs come into contact with TLR-expressing cells through several different mechanisms. Direct cellular damage from oxidative stress during ischemia results in the passive release of DAMPs from necrotic cells; additionally, they are liberated from the cell matrix by proteases. Lastly, it appears that DAMP release from stressed cells may be a regulated mechanism. Recent reports show that DAMP-mediated TLR activation itself regulates the additional release of a number of danger signals, and therefore TLR signaling may function in an autocrine/paracrine fashion that culminates in excessive innate immune activation and organ damage.

## 5. TLR4 in Liver I/R

**Figure 1:**
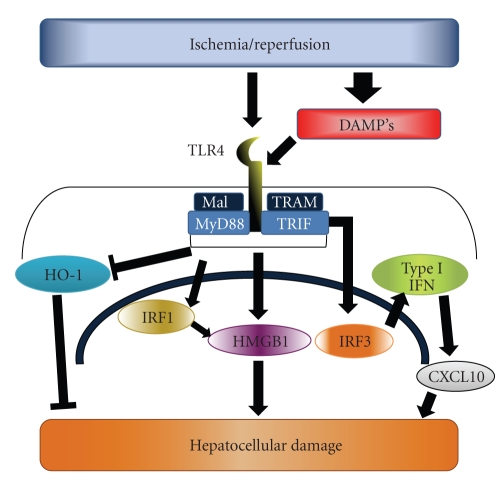
Role of TLR4 in hepatic I/R injury. Signaling through the pattern recognition receptor TLR4 mediates multiple inflammatory pathways following hepatic I/R. The activation of TLR4 signaling is dependent, in part, on circulating DAMPs. Downstream signaling events include the TRIF-dependent activation of IRF3, the production of Type I IFN, and upregulation of CXCL10. Additionally, TLR4 activation of IRF1 promotes release of the DAMP, HMGB1. Lastly, TLR4 activation is thought to suppress the cytoprotective HO-1 pathway.

Amongst the most studied TLR in hepatic I/R is TLR4. Buetler et al. were the first to discover that TLR4 is a sensor for bacterial lipopolysaccharide (LPS) [27], and subsequent studies showed that TLR4 also plays a role in a number of acute sterile injury models, including liver I/R (Figure 1) [28]. 

### 5.1. Warm I/R

The involvement of TLR4 in warm I/R injury was first described by Wu and colleagues in 2004. This study used a model of partial hepatic I/R and showed that mice deficient in TLR4 signaling experienced significantly less liver damage compared to their wild-type counterparts. TLR4-deficient mice had significantly lower levels of serum aspartate transaminase levels (AST), as well as decreased levels of TNF-*α* mRNA and myeloperoxidase (MPO) after I/R [29]. Other groups have subsequently published similar findings, all of which show that TLR4-deficient mice experience less injury and inflammation after warm I/R [30–33]. These studies provided clues that TLR4 activation during I/R promotes damage through secretion of cytokines and recruitment of inflammatory cells to the liver. In addition to reduced secretion of proinflammatory mediators, Shen et al. also found that the protective Heme Oxygenase-1 (HO-1) pathway was upregulated in TLR4 deficient mice, suggesting that suppression of this pathway downstream of TLR4 activation is another damage-promoting mechanism during I/R [31]. Another model of sterile, ischemic injury is hemorrhagic shock (HS). HS results in systemic hypoperfusion and I/R-like damage to the liver. In addition to data from I/R models, we have shown that the liver damage induced by hemorrhagic shock is also strongly TLR4-dependent [34], suggesting a common mechanism between these two ischemic insults.

One important question from early studies was the agent(s) responsible for activating TLR4 after I/R. While TLR4 is capable of recognizing a number of substrates, our laboratory has shown a key role for the endogenous nuclear molecule HMGB1 [32]. Administration of recombinant HMGB1 prior to I/R resulted in a significant increase in hepatocellular damage in TLR4 WT but not TLR4-deficient mice. Conversely, treatment with a neutralizing antibody to HMGB1 provided significant protection from I/R damage in WT mice but afforded no further protection from damage in TLR4-deficient mice. While this study showed that HMGB1 is capable of activating TLR4 in the setting of warm hepatic I/R, we subsequently found that TLR4 activation actively regulated the release of HMGB1 from hepatocytes. These studies showed that circulating levels of HMGB1 were significantly lower in TLR-defective mice after I/R, and we found this phenomenon to be dependent on TLR4-dependent production of ROS and Calcium/Calmodulin-Dependent Protein Kinase (CaMK) signaling [35]. Thus, while HMGB1 is an activator of TLR4, its release is also determined, in part, by TLR4 itself.

TLR4 is expressed in numerous cell types in the liver, including parenchymal hepatocytes and bone marrow-derived immune cells. In a study to delineate the role of TLR4 in different cell types of the liver, we generated TLR4 chimeric mice and found that hepatic injury after I/R is largely dependent on TLR4 expression on bone-marrow derived cells [36]. A chimeric mouse model in which recipient mice received lethal irradiation to eradicate bone marrow cells, followed by bone marrow transplantation, was used. This procedure permits reconstitution of bone marrow with syngeneic bone marrow from mice with either functioning or mutant TLR4 signaling. After 8–10 weeks, the immune cells within the liver are replaced with cells expressing the new phenotype while the long-lived parenchymal cells retain the host's phenotype. WT mice that lacked TLR4 on bone marrow-derived cells were protected from I/R injury similar to mice that lacked TLR4 in both parenchymal and bone marrow-derived cells. In contrast, transfer of WT TLR4 bone marrow cells to Mutant TLR4 mice resulted in significantly increased hepatocellular injury after I/R. In addition, WT mice, but not Mutant TLR4 mice, were protected from I/R injury following phagocytic cell depletion with gadolinium chloride while overexpression of dendritic cells with plasmid GM-CSF worsened damage in WT, but not Mutant, TLR4 mice [37]. Taken together, these data indicate that TLR4 signaling in NPCs, such as Kupffer and dendritic cells, is required for I/R-induced injury and inflammation. Similar results were reported in another study by Hui et al., who reported that TLR4 expression on both bone marrow and parenchymal cells was necessary for maximal damage after I/R. Chimeric mice that lacked TLR4 on either bone marrow-derived or parenchymal cells were protected from injury compared to mice with functional signaling in both cell populations. This study also showed that functional TLR4 signaling on nonbone marrow-derived cells (sinusoidal endothelial cells and hepatocytes) was necessary for the expression of ICAM-1, which adheres to circulating neutrophils recruited to the liver following damage. Neutrophil infiltration itself, however, was found to be dependent on bone marrow-derived cells [38]. 

Downstream TLR4 signaling pathways include both the MyD88 and TRIF signaling cascades, and TLR4-mediated hepatocellular damage appears to be independent of MyD88 signaling. This insight came from a study by Zhai et al. who used MyD88 KO and IRF3 KO mice. MyD88 KO mice were used to study one branch of the TLR4 signaling cascade while IRF3 KO mice were used to study TRIF-dependent signaling, since IRF3 is downstream of TRIF. They found that protection was not conferred to MyD88 KO mice during I/R while IRF3 KO mice were significantly protected [39]. These results suggested that TRIF-dependent signaling was critical in mediating warm I/R damage. Since TRIF-dependent signaling activates the interferon response, these authors undertook additional studies to delineate downstream mediators and found that both Type 1 Interferons and CXCL10 were critical for I/R damage in a TRIF-dependent fashion [40, 41]. This work suggests TLR4 mediated damage is MyD88 independent and involves activation of IRF3, type I interferons, and production of CXCL10. 

Another member of the interferon regulatory factor family, IRF1, has also been shown to be downstream of TLR4 activation [42], and this molecule is important for I/R injury. IRF1 KO mice are protected from hepatocellular damage in warm I/R while *in vivo* adenoviral overexpression of IRF1 augments damage [43]. Furthermore, two studies using orthotopic liver transplantation have shown that IRF1 expression in hepatocytes is critical for mediating I/R injury [44, 45]. Unpublished work from our group shows that IRF1 expression during I/R is TLR4 dependent, as TLR4-defective mice have decreased levels of IRF1 expression after I/R. *In vitro*, IRF1 expression was decreased in TLR4-deficient hepatocytes, and adenoviral transfection of WT TLR4 into these cells restored IRF1 expression. We also found that IRF1 expression is critical for the release of the danger signal HMGB1. We observed that serum levels of HMGB1 in IRF1 KO mice were significantly lower than their wild-type counterparts, and IRF1 KO hepatocytes release significantly less HMGB1 into cell culture supernatants after nonlethal hypoxic stimuli. These novel findings connect three important mediators of I/R injury, TLR4, IRF1, and HMGB1.

### 5.2. Cold I/R

TLR4 has also been implicated in cold I/R injury during liver transplantation. Tsoulfas and colleagues showed that components of the LPS signaling pathway were inovolved in hepatic transplant preservation injury [46]. Subsequently, Shen et al. used an orthotopic liver transplant (OLT) model and found that absence of TLR4 signaling in donor livers protected recipients from hepatocellular damage. A similar pattern of protective changes observed in TLR4 KO grafts was observed in this model compared to similar studies in warm I/R. These changes include decreased serum ALT, CXCL10, ICAM-1, TNF-*α*, IL-1*β*, IL-2, and IFN*γ* and an increase in protective HO-1. These effects were observed regardless of the TLR4 genotype of the recipient [47]. In addition to murine studies, increased expression of TLR4 on monocytes of liver transplant patients has also been found correlate with acute rejection [48].

## 6. TLR2 in Liver I/R

 Although the role of TLR2 in I/R is less well delineated, a number of studies have made important strides in determining its role. Classic TLR2 ligands include PAMPs from gram positive bacteria including peptidoglycan and lipoteichoic acid. Zhai and colleagues examined TLR2 KO mice in a model of warm I/R and found no significant difference in serum ALTs in TLR2 KO mice compared to WT controls [30]. Similar findings were noted by Shen and colleagues, who also showed that TLR2 KO mice were not afforded protection in warm hepatic I/R [31]. While no studies in hepatic I/R have identified a significant role for TLR2 in promoting or preventing damage, it has been implicated in other models of I/R, namely cardiac [49, 50], renal [51, 52], brain [53–55], and gut [56]. Taken together, these studies support the possibility that tissue and cell-type specific roles for TLRs exist.

## 7. TLR9 in Liver I/R

 TLR9 is an intracellular molecule that functions as sensor for DNA. It was originally described by Hemmi et al. who showed that TLR9 KO mice failed to respond to bacterial CpG DNA [57]. Upon internalization of DNA from the plasma membrane, TLR9 translocates from the ER to endosomes and binds DNA. Although it was originally thought that TLR9 was specific for the recognition of bacterial DNA, it was subsequently discovered that host DNA is also capable of activating TLR9 [58–61]. In regard to liver I/R injury, Bamboat et al. found that TLR9 KO mice were protected from injury in warm I/R by a mechanism dependent on TLR9 expression on liver nonparenchymal cells. Using chimeric mice generated from adoptive bone marrow transfer, they reported that TLR9 expression on bone marrow-derived cells, but not liver parenchymal cells, to be important for damage after I/R [62]. *In vitro* experiments with hepatocytes and NPCs derived from TLR9 WT and KO mice suggested that DNA derived from necrotic hepatocytes was capable of activating TLR9-competent NPCs, causing increased production of proinflammatory cytokines. In contrast to its overall effect in liver I/R, a recent report from the same group showed that secretion of the anti-inflammatory cytokine IL-10 from conventional dendritic cells (cDCs) during liver I/R is TLR9 dependent. Depletion of cDCs resulted in increased liver damage after I/R, and this phenomenon could be reversed by exogenous transfer of WT cDCs prior to I/R. However, transfer of TLR9−/− cDCs failed to rescue mice from increased damage, suggesting that TLR9 activation in cDCs is protective [63]. These findings are in contrast to a report by our group, which demonstrated that DCs promote I/R injury through a TLR4-dependent pathway involving HMGB1 [37]. The seemingly conflicting roles of liver DC in liver I/R may be due to differences in the definition of DCs used in these studies. DC markers such as CD11c may be expressed in a wide variety of both intrahepatic leukocytes and nonhematopoietic cells [64, 65]. Thus, the role of various TLRs on DCs in modulating the hepatic microenvironment following I/R remains to be fully elucidated. These future studies will require stricter validation to determine that the cells being analyzed are indeed morphologically DCs.

## 8. Conclusion

**Figure 2:**
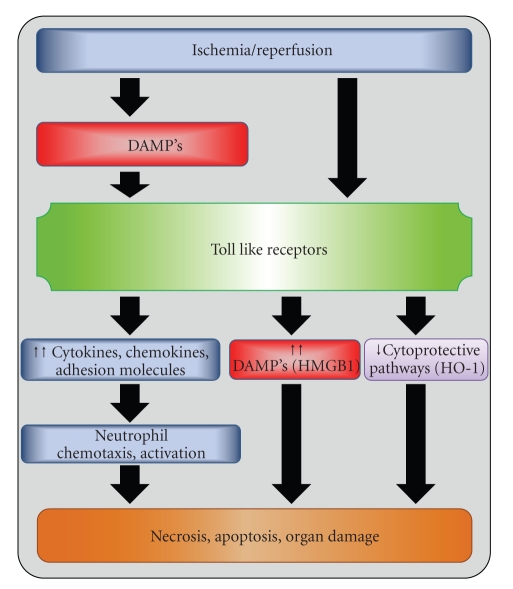
TLRs coordinate the response to hepatic I/R injury. Toll-like receptors are proximal to most elements of hepatic I/R injury. Following an ischemic insult, TLR activation by circulating DAMPs sets off signaling cascades in multiple cell types that coordinates the inflammatory response. Both TLR4 and TLR9 activation on hepatic nonparenchymal cells promotes production of proinflammatory cytokines, leading to additional neutrophil recruitment and organ damage. TLR4 activation has also been shown to augment the release of circulating DAMPs and may suppress protective cellular pathways.

Taken together, the results of the studies summarized in this paper provide convincing evidence demonstrating that TLR signaling is involved in the early activation of the innate immune system in the setting of I/R injury. Accumulating evidence points to endogenous molecules that are released from stressed or damaged cells or tissues during the course of I/R, as important triggers of the immune response in the setting of I/R (Figure 2). While much of the early work focused on the role of TLR4, new and future work will continue to delineate the roles of other TLRs in I/R injury. A better understanding of the molecular interactions involved in these processes, as well as greater knowledge of the intracellular pathways that mediate these signaling cascades, may ultimately allow for the development of therapeutics aimed at ameliorating I/R injury and its adverse consequences.
